# Molecular dissection of an adaptive epigenetic memory mechanism in norway spruce

**DOI:** 10.1186/1753-6561-5-S7-O25

**Published:** 2011-09-13

**Authors:** Igor Yakovlev, YeonKyeong Lee, Björn Rotter, Tore Skrøppa, Jorunn Elisabeth Olsen, Øystein Johnsen, Carl Gunnar Fossdal

**Affiliations:** 1 Norwegian Forest and Landscape Institute, PO box 115, 1431, Ås, Norway; 2 Norwegian University of Life Sciences, Department of Plant and Environmental Sciences, 1432, Ås, Norway; 3 GenXPro GmbH, Franksfurter Innovationszentrum (FIZ), Altenhöferallee 3, 060438 Franskfurt am Main, Germany

## 

In Norway spruce, environmental conditions during the reproduction can greatly influence progeny performance. We found that the temperature during post meiotic megagametogenesis (zygotic embryogenesis) and seed maturation shift the growth cycle program of the embryos in the seeds, resulting in significant and long lasting phenotypic changes in the progeny. Traits that are affected include the timing of dehardening and bud burst in the spring; leader shoot growth cessation in the summer, and bud set and cold acclimation in the autumn. All processes are advanced or delayed in correspondence with the temperature during female reproduction. Colder reproductive environment advance bud set and cold acclimation during autumn and dehardening and bud burst during spring in their progenies. Temperature dependent difference in timing of terminal bud formation in identical clones was equivalent to a 4–6° latitudinal ecotypic difference. The progeny “remember” the temperatures and photoperiod prevailing during zygotic embryogenesis and seed maturation and this memory, affecting the climatic adaptation in this species, is an epigenetic phenomenon.

This phenomenon is not only of evolutionary significance but has clear practical implications. This memory can help the conifer to cope with the anticipated rapid change in climatic conditions. It will have importance for the deployment of seedlings produced in seed orchards containing clones that are translocated to warmer sites, and it may be used to produce seedlings that have specific adaptive properties. So, it is possible to produce distinct phenotypes (epitypes) in Norway spruce, however this type of long lasting effects is not well documented in other organisms so far.

The molecular mechanism behind this striking epigenetic memory phenomenon is not yet unraveled but transcriptional changes are clearly involved. In epigenetically different progenies, transcriptional analysis revealed that seedlings from full-sib families produced at different embryogenesis temperature under long and short day conditions differed. Suppressive subtracted cDNA libraries revealed significant differences in their transcriptomes. Using qRT-PCR, microRNA pathways genes *PaDCL1* and *2* and *PaSGS3* as well as transposons related genes are differential expressed in the epigenetically different progenies with phenotypic differences in bud burst and bud set.

MicroRNAs (miRNAs) are endogenous small RNAs that can exert epigenetic gene regulatory impacts. We have examined the possible role of miRNA in the epigenetic phenomena, and found that Norway spruce contains a set of conserved miRNAs as well as a large proportion of novel non-conserved miRNAs. From concatemerized small RNA libraries from seedlings from the same parents, originated from seeds developed in a cold and warm environment from a family with distinct epigenetic effects, contrasted to one from a family with little response, miRNAs potentially involved in this epigenetic memory was identified. Most of the miRNAs target unknown genes or genes with no known function. The expression of seven conserved and nine novel miRNAs showed significant differences in transcript levels in progenies with distinct epigenetic difference in bud set, but not in the progenies from a non-responding family, making them excellent candidate miRNAs. The differentially expression of specific miRNAs in genetically identical but epigenetically different progeny indicate their putative participation in the epigenetic regulation.

Epigenetic mechanisms influence phenotype through altered regulation of gene expression that is mitotically (and sometimes meiotically) propagated. Understanding the mechanisms involved in the initiation, maintenance, and heritability of epigenetic states is an exiting aspect of research in current biology. Epigenetic regulation may be realized through several interconnected molecular pathways including DNA methylation, histone modification and chromatin remodeling, small non-coding RNAs and transposable element regulation.

Among spruce ESTs we found 64 homologs of genes described as involved in DNA methylation, histone modification and chromatin remodeling and small RNA biogenesis in other plant species. In general, known epigenetic mechanism related genes are very well represented in the spruce genome. We analyzed the transcription patterns of these genes using RT-PCR in epigenetically different zygotic embryogenic samples on different stages of development and in seedlings, originated from full-sib families clearly differed in epigenetic response. The largest difference in gene expression of selected genes was found at the earlier stages of embryogenesis while in seedlings a low number of these genes were differentially expressed. Most of the known epigenetic mechanism related genes in seedlings were steadily expressed in all studied samples independently of their epitype.

To get a deeper analysis of epigenetic related transcriptome we used high-throughput sequencing (RNA-seq and miRNA-seq) in cooperation with GenXPro GmbH. Using MACE (massive cDNA 3’end sequencing) deep mRNA sequencing on the Illumina GSII platform, we analyzed the genes differentially expressed in *P. abies* during early stages of embryonic development. We selected genes which could be involved into epigenetic response by comparison warm and cold originated “embryonic epitypes” from the same full-sibs family somatic embryos developed in cold (18°C) and warm (30°C) environmental conditions. Additionally, for more distinct analysing of the large amount of “no database hit” reads we sequenced one normalised library using 454 Titanium GS FLX sequencing to get reference transcript set of expressed genes. The sequencing data is currently under processing and we are going to discuss main results here.

To proceed with our initial study of miRNAs in spruce, we used Illumina/SOLEXA sequencing to identify small RNAs expressed at the same epigenetic responsive family developed in warm and cold environment progenies following short-day treatment. The identification of novel miRNA candidates are in progress and the confirmation of conserved and novel miRNA by qRT-PCR analysis will be presented.

